# Deep Vein Thrombosis and Pulmonary Embolism Among Patients With a Cryptogenic Stroke Linked to Patent Foramen Ovale—A Review of the Literature

**DOI:** 10.3389/fneur.2020.00336

**Published:** 2020-05-05

**Authors:** Annaelle Zietz, Raoul Sutter, Gian Marco De Marchis

**Affiliations:** ^1^Department of Neurology, University Hospital Basel and University of Basel, Basel, Switzerland; ^2^Department of Intensive Care, University Hospital Basel and University of Basel, Basel, Switzerland

**Keywords:** cryptogenic stroke, patent foramen ovale, deep vein thrombosis, pulmonary embolism, venous thromboembolism

## Abstract

**Background:** Venous thromboembolism (VTE) can occur simultaneously with a cryptogenic stroke (CS) linked to patent foramen ovale (PFO), given paradox thromboembolism as potential stroke cause. However, little is known on the frequency of concomitant VTE and CS. We aimed to review the literature on the frequency of VTE in patients with CS linked to PFO (primary aim) and of ischemic stroke (IS) among patients with pulmonary embolism (PE) (secondary aim).

**Methods:** We performed a Medline search for cohort studies, written in English, with the following characteristics: (a) enrolling patients hospitalized for an acute ischemic stroke undergoing a work-up for deep venous thrombosis (DVT) and/or PE. To be included in this review, a study had to have at least a subgroup of patients with PFO; (b) the time interval between the index stroke and the work-up had to be within 40 days and the studies had to differentiate between DVT and PE. For the secondary aim, studies had to include patients with acute PE, known PFO-status and routine brain imaging on admission or within 1 year.

**Results:** We found eight studies reporting on the frequency of VTE after an acute CS linked to PFO. Concerning DVT, the reported frequency ranged between 7 and 27%; concerning PE, it lied between 4.4 and 37%. Six studies assessed the frequency of ischemic brain lesions among patients with an acute PE. In all studies, the presence of PFO was associated with ischemic brain lesions, both at baseline and follow-up.

**Conclusion:** VTE can be detected in patients with CS linked to PFO. While –based on the presented literature–routine screening for VTE in patients with CS linked to PFO does not appear justified, history taking, and clinical exam should consider concomitant VTE. Whenever clinically suspected, the threshold to trigger ancillary testing for VTE should be low. Among patients with an acute PE and PFO, vigilance for new neurologic deficits should be increased, with a low threshold for brain imaging.

## Background

Up to date, ~25% of ischemic stroke are described as cryptogenic (CS) (1). Even though a prospective follow up study did not describe a PFO as an independent risk factor for ischemic stroke in general (2), various studies demonstrated an association between PFO and CS (3–6). The suspected pathophysiological mechanism is paradox embolism, enabling a passage of the venous thrombus through the PFO into the arterial circulation (7). Imaging demonstrating the migration of a thrombus was also described (8). The source of venous thromboembolism (VTE) is often suspected in the peripheral venous system. An acute rise of the right atrial pressure—for example through a Valsalva maneuver—could facilitate the passage through a PFO. Ozcan et al. (9) described an association between Valsalva maneuver and a history of VTE with a PFO related ischemic stroke. Four trials demonstrated—after PFO closure—a reduced incidence of recurrent ischemic stroke compared to antithrombotic therapy (antiplatelet or anticoagulation) (10–13). However, none of the trials mandated screening for VTE, and all had anticoagulation as an exclusion criterion. In clinical practice, detection of VTE leads to anticoagulation, potentially postponing PFO closure as long as anticoagulation is needed, given the lack of data on concomitant anticoagulation linked to PFO closure.

In addition, patients with PFO and a diagnosed PE may be at increased risk for ischemic stroke, further underlying the role of paradox embolism (14).

In this work, we aim to review the literature on the frequency of VTE in patients with CS linked to PFO, and the frequency of ischemic stroke in patients with PE.

## Methods

For this narrative review, we performed a Medline search using the keyword “deep vein thrombosis,” “patent foramen ovale” and “ischemic stroke.” Two reviewers (AZ, GMDM) evaluated the included studies. We searched for cohort studies, written in English after 1990, enrolling patients hospitalized for an acute ischemic stroke undergoing a work-up for deep venous thrombosis (DVT) and/or pulmonary embolism (PE). To be included in this review, a study had to have at least a subgroup of patients with PFO and had to differentiate between DVT and PE. The time interval between the index stroke and the work-up did not have to exceed 40 days, to increase chances of finding VTE linked to paradox embolism rather than secondary to immobilization due to the index stroke.

Concerning the secondary aim, we included cohort studies written in English who (a) enrolled patients with acute pulmonary embolism (b) performed a search for patent foramen ovale and (c) carried out a brain imaging after the diagnosis of an acute PE. In our Medline search we used the keyword “patent foramen ovale,” “pulmonary embolism” and “stroke.”

## Results

Our review identified eight studies reporting the frequency of VTE in patients with CS linked to PFO. Six of these studies did not compare the frequency of DVT between CS and non-CS patients (Table 1) (15–20), two studies did (Table 2) (22, 23).

**Table 1:** Summary of studies on the frequency of DVT/PE in patients with CS linked to PFO.

**References**	**Patient with CS linked to PFO: % of the whole cohort (n)**	**Work-up for VTE**	**Days between Index stroke and VTE work-up**	**Frequency of DVT/PE in patients with CS linked to PFO**
Lethen et al. (15)	23% (*n* = 53)	Venography	8 ± 3	DVT: 9.5% (5/53) PE: N/A
Cramer et al. (16)	100% (*n* = 37)	VenographyMRV	8	DVT: 27% (10/37) PE: N/A
Lapergue et al. (17)	100% (*n* = 114)	Combined CT-Venography and pulmonary angiography	4–9	VTE: 10.5% (12/114) DVT: 8.8% (10/114) Silent PE: 4.4% (5/114)
Osgood at al. (18)	100% (*n* = 50)	MRV	4 ± 3	DVT: 8% (*n* = 4) May Thurner Syndrom*: 10% (*n* = 5) PE: N/A
Tanislav et al. (19)	100% (*n* = 151)	Ventilation perfusion scintigraphy	N/A	DVT: 7% (*n* = 11) Silent PE: 37% (*n* = 56)
Ranoux et al. (20)	19.1% (*n* = 13)	Venography	0–38	DVT: 8% (*n* = 1) in a plegic leg 14 days after index stroke PE: N/A

PFO, patent foramen ovale; CS, cryptogenic stroke; PE, pulmonary embolism; DVT, deep vein thrombosis; VTE, venous thromboembolism; MRV, magnetic resonance venography; N/A, not available. *May Thurner Syndrome indicates an anatomical variation, in which the origin of left V. iliaca communis is being anatomically narrowed by the right A. iliaca communis. This reduces venous blood flow, increasing the risk of DVT (21).

**Table 2:** Summary of studies comparing the frequency of DVT among patients with cryptogenic vs. known-cause stroke.

**References**	**Population % (n)**	**Work-up for DVT**	**DVT prevalence in Non-CS vs. CS, % (n)**	**Time between Index stroke and DVT workup**
Cramer et al. (22)	Non-CS: 52% (*n* = 49) CS: 48% (*n* = 46, among them 61% with an PFO or ASD)	MRI Venogramm	Total patients 4% (2/49) vs. 20% (9/46); *p* = 0.025 Subgroup with PFO 0% (0/9) vs. 21% (6/28); *p* = 0.30	48.9 ± 16.1 h
Liberman et al. (23)	All Patients had PFO (*n* = 131) CS: 74.8% (*n* = 98) Non-CS: 25.2% (*n* = 33)	MRI Venogramm LE duplex ultrasound	9.1% (3/33) vs. 7.2% (7/98); *p* = 0.71	0–4 days

*PFO, patent foramen ovale; CS, cryptogenic stroke; PE, pulmonary embolism; DVT, deep vein thrombosis; VTE, venous thromboembolism; MRV, magnetic resonance venography; N/A, not available*.

### Studies Not Comparing the Frequency of DVT in Patients With CS Vs. Non-CS

Investigation regarding the emergence of VTE were performed within 0 to 38 days after index stroke. Concerning DVT, the reported frequency ranged between 7 and 27%; concerning PE, it lied between 4.4 and 37% (15–20). Concomitant DVT in patients with PE were described in two studies: Lapergue et al. (17) found a DVT in 3 out of 5 patients with silent PE, Tanislav et al. (19) in 8 out of 56 patients. In a study by Osgood et al. (18) four pelvic DVT were diagnosed (8%), as well as 5 cases of May Thurner Syndrom. The latter describes an anatomical variation, in which the left V. iliaca communis is being anatomically narrowed by the right A. iliaca communis. This reduces venous blood flow, increasing the risk of DVT (21).

### Studies Comparing the Frequency of DVT in Patients With CS Vs. Non-CS

The prospective PELVIS study found—in patients with CS—more MR-venograms with pelvic DVT compared to non-CS (20 vs. 4%, *p* = 0.025), suggesting the source of paradox embolism may be located in the pelvic veins in a subset of patients with CS. Notably—when looking at the subgroup with PFO—there was no significant difference between CS and non-CS in the frequency of DVT (21 vs. 0%, *p* = 0.30) (22). In the retrospective study of Liberman et al. (23), contrast enhanced MR-venograms were used, and patients with CS vs. non-CS were compared. All patients, both CS and non-CS, had PFO. No significant difference in the frequency of DVT—both pelvic and lower extremity—was found between CS and non-CS (7.2 vs. 9.1%, *p* = 0.71), calling for further research before implementing routine pelvic MR-venograms. Clinical evidence of a PE was found in one patient with chronic lower extremity DVT.

### Studies on the Frequency of Ischemic Strokes in Patients With Acute PE and PFO

We found six studies; detailed analyses regarding population characteristics, diagnostic measures and time to interventions after admission are outlined in Table 3 (14, 24–28). Overall, ischemic stroke was reported to be diagnosed within 2–22 days following after admission and was more frequent in patients with overt PFO with four studies revealing statistical significance (14, 25, 27, 28). In the study of Konstantinides et al. (27) all investigations were performed during the hospital stay (22 ± 17 days).

**Table 3:** Frequency of ischemic brain lesions among patients with an acute PE, with or without PFO.

**References**	**Study population**	**Diagnostic**	**Frequency of ischemic brain injuries (PFO vs. Non PFO)**	**Days to intervention after admission**
Le Moigne et al. (24)	Acute PE (*n* = 315): • PFO (*n* = 42) • Non PFO (*n* = 273)	cMRI TTE	Silent or symptomatic IBL 21.4% (9/42) vs. 5.5% (15/273) Symptomatic IBL 9.5% (4/42) vs. 1.5% (4/273) CS 16.7% (7/42) vs. 1.8% (5/273)	cMRI and TTE: 7 days
Vindiš et al. (25)	Acute PE (*n* = 78): • PFO (*n* = 31) • Non PFO (*n* = 47) 12 month follow-up (*n* = 58)	cMRI TTE/TEE	At Baseline 64.5% (20/31) vs. 40.4% (19/47); *p* = 0.06 At follow up New IBL 33.3% (7/21) vs. 5.4% (2/37); *p* = 0.008	TEE and TTE baseline TTE: 12 month follow up cMRI (baseline, 12 month follow up)
Doyen et al. (26)	Intermediate risk PE (*n* = 41) • PFO (*n* = 23) • Non PFO (*n* = 18)	cMRI TTE/TEE	17.1% (*n* = 7) (PFO in all cases, 30.4% with PFO had an IBL)	TTE/TEE: 1–3 days cMRI: 5 ± 4 days
Clergeau et al. (14)	Acute PE (*n* = 60) • PFO (*n* =15) • Non PFO (*n* = 45)	cMRI TTE	33.3% (5/15) vs. 2.2% (1/45) *p* = 0.003	cMRI: 3 ± 1 days
Konstantinides et al. (27)	Acute PE (*n* = 139) • PFO (*n* = 48) • Non PFO (*n* = 91)	cCT or Autopsy	13% (6/48) vs. 2.2% (2/91), *p* = 0.02	22 ± 17 days
Goliszek et al. (28)	Acute PE (*n* = 55) • PFO(*n* = 19) • Non PFO (*n* = 36)	cMRI TTE	21% (4/19) vs. 0% (0/36) *P* = 0.02	cMRI: 4.91 ± 4.1 days TTE: N/A

*PFO indicates patent foramen ovale; PE: pulmonary embolism; cMRI: cranial magnetic resonance imaging; TTE: transthoracic echocardiography; TEE: transesophageal echocardiography; IBL: ischemic brain lesions; N/A: not available*.

## Discussion

Studies demonstrated a wide range in the reported frequency of VTE in patients with CS linked to PFO, likely because the diagnostic of lower extremity DVT depends on the investigator and expertise in using duplex sonography (15, 20). In asymptomatic patients, a lower sensitivity (60%) of venous duplex sonography is described (29).

The two studies comparing the frequency of DVT between patients with CS vs. non-CS yielded conflicting results. In PELVIS (22)—but not in the study by Liberman et al. (23)—a higher frequency of DVT was observed among patients with CS than among those with non-CS. Differences in the DVT screening protocols as well as baseline characteristics may explain the conflicting results. In contrast to PELVIS, in the study of Liberman et al. (23) MR-venograms were contrast-based (i.e., less prone to artifacts), all patients had PFO, were older (mean age 46 years vs. 57 years, respectively) and had a higher burden of cardiovascular risk factors. To note, neither the subgroup of PFO patients in the PELVIS study nor the patients in the study of Libermann at al. (23) showed significant differences on the DVT frequency. Before implementing routine MR-Venography in clinical practice, further research is needed.

Liberman et al. (23) used the Causative Classification System to retrospectively classify the etiology of the ischemic stroke. Of note, patients with transient ischemic attacks were also included. In PELVIS, a stroke neurologist was responsible to identify and classify the cause of the ischemic stroke, based on the TOAST criteria.

In the three pivotal trials on PFO-closure (10, 11, 13), a search for VTE was not part of the routine diagnostic work up. In the follow up examinations, the occurrence of PE or DVT in the PFO closure group and the medical therapy group were reported as adverse events. Suspecting the frequency of underdiagnosed VTE, the risk of PE could even rise after PFO closure and without an effective oral anticoagulation. However, only the long-term evaluation of the RESPECT trial showed a higher detection rate of PE in the PFO closure group (24% vs 0.6%, *p* = 0.03) (12).

The CLOSE study compared PFO closure to oral anticoagulation. Three recurrent ischemic strokes were reported in the oral anticoagulation arm, whereas no recurrent stroke was described in the PFO closure arm (30). Since no trial allowed for PFO closure under concomitant oral anticoagulation, there are no data concerning PFO closure under oral anticoagulation. Thus, the diagnosis of DVT/PE—indicating oral anticoagulation for at least 3 months—could postpone PFO-closure leaving patients at risk of a stroke recurrence even under oral anticoagulation. To note, the early start of an oral anticoagulation could also lead to hemorrhagic transformation (7).

The reported association between PE and IS in patients with PFO further underlines the role of paradox embolism. Particularly in patients with intermediate-risk PE, PFO related ischemic brain lesions were frequent, up to 17.1% (26). Of note, none of these patients had a significant carotid stenosis or suspected cardioembolic source of ischemic stroke. Even under effective oral anticoagulation, Vindiš et al. (25) reported a significant difference in recurrent ischemic lesions in patients with PFO after PE, raising the question if PFO closure should be considered in some patients with PE (25).

## Conclusion and Clinical Implications

Since VTE calls for therapeutic anticoagulation, the clinically important question arises if a baseline search for DVT in patients with CS linked to PFO is necessary. The reported frequency of DVT in two studies using MRI Venogram showed a large range of up to 20% (22, 23) while other studies described lower frequencies (15, 17). In patients with CS linked to PFO, the focus of medical history and physical exam should be intensified on the search for DVT/PE. The threshold for DVT/PE screening should be low, giving the potential subsequent indications for oral anticoagulation linked to PFO screening. Further prospective studies are needed to establish the optimal diagnostic work up for VTE/PE in patients with CS linked to PFO, as well as the safety of combining anticoagulation to PFO-closure.

## Data Availability Statement

All datasets generated for this study are included in the article/supplementary material.

## Author Contributions

GD formulated the research question. AZ and GD summarized and extracted the data manually from published papers for this review. GD, AZ, and RS drafted the article and reviewed it critically.

## Conflict of Interest

The authors declare that the research was conducted in the absence of any commercial or financial relationships that could be construed as a potential conflict of interest.
